# Co-Expression of NEU2 and GBA3 Causes a Drastic Reduction in Cytosolic Sialyl Free *N*-glycans in Human MKN45 Stomach Cancer Cells—Evidence for the Physical Interaction of NEU2 and GBA3

**DOI:** 10.3390/biom5031499

**Published:** 2015-07-16

**Authors:** Li Wang, Junichi Seino, Haruna Tomotake, Yoko Funakoshi, Hiroto Hirayama, Tadashi Suzuki

**Affiliations:** 1 Glycometabolome Team, Systems Glycobiology Research Group, RIKEN-Max Planck Joint Research Center for Systems Chemical Biology, RIKEN Global Research Cluster, Saitama 351-0198, Japan; E-Mails: wangli0909@gmail.com (L.W.); jseino@riken.jp (J.S.); haruna.tomotake@riken.jp (H.T.); yfunakos@sbigroup.co.jp (Y.F.); hiroto-hirayama@riken.jp (H.H.); 2 Graduate School of Science and Engineering, Saitama University, Saitama 338-8570, Japan

**Keywords:** free *N-*glycans, GBA3, glycan catabolism, NEU2, sialyl oligosaccharides

## Abstract

It is well known that the “free” form of glycans that are structurally related to asparagine (N)-linked glycans (“free *N*-glycans”) are found in a wide variety of organisms. The mechanisms responsible for the formation/degradation of high mannose-type free *N*-glycans have been extensively studied in mammalian cells. Recent evidence, however, also suggests that sialylated, complex-type free *N*-glycans are also present in the cytosol of various mammalian-derived cultured cells/tissues. We report herein on an investigation of the mechanism responsible for the degradation of such sialyl free *N*-glycans. The findings show that the amount of glycans is dramatically reduced upon the co-expression of cytosolic sialidase NEU2 with cytosolic β-glycosidase GBA3 in human stomach cancer-derived MKN45 cells. The physical interaction between NEU2 and GBA3 was confirmed by co-precipitation analyses as well as gel filtration assays. The NEU2 protein was found to be stabilized in the presence of GBA3 both *in cellulo* and *in vitro*. Our results thus indicate that cytosolic GBA3 is likely involved in the catabolism of cytosolic sialyl free *N*-glycans, possibly by stabilizing the activity of the NEU2 protein.

## 1. Introduction

The *N*-glycosylation of proteins constitutes one of the most abundant co- or post-translational modifications of eukaryotic proteins, and numerous studies have provided evidence for the pivotal roles of this modification in modulating the physicochemical/physiological properties of carrier proteins [1,2,3,4]. While most, if not all, of the biosynthetic pathway for *N-*glycosylation has been well clarified in mammalian cells, the issue of how these glycans are degraded in cells remains relatively unclear, except for the degradation process that occurs in lysosomes [5,6]. For example, while the occurrence of free oligosaccharides that are structurally related to *N*-glycans (free *N*-glycans; FNGs) as products of the “non-lysosomal” catabolic pathway for glycoconjugates has been well documented in mammalian cells or yeast [7,8], most of the studies have been limited to high mannose–type FNGs. On the other hand, the occurrence of sialylated, complex-type FNGs has been reported in the cytosol of mouse liver [9], human stomach cancer-derived MKN7/45 cells [10], mice autophagy-defective cells [11] as well as in human cancer tissues [12,13], whereas the issue of how these glycans are formed and degraded remains poorly understood.

It has been previously documented that, when cytosolic sialidase NEU2 is expressed in MKN45 cells, the levels of sialyl FNGs become decreased [10]. As the accumulation of non-sialylated, complex-type FNGs was not obvious in these cells, we hypothesized that there must be a specialized catabolic pathway that processes non-sialyl complex-type FNGs. A literature survey prompted us to examine GBA3, a cytosolic β-glycosidase that is produced by mammals [14,15,16]. This glycosidase exhibits a broad substrate specificity, and therefore it has been hypothesized that this enzyme may play a role in hydrolyzing xenobiotic glycosides [17]. It was subsequently reported that this enzyme also appears to be involved in the nonlysosomal catabolic pathway for glucosylceramide [18].

In this study, we report on the possible role of GBA3 in the catabolism of cytosolic sialyl FNGs in MKN45 cells. To our surprise, the co-expression of NEU2 and GBA3 resulted in the nearly complete loss of sialyl FNGs in MKN45 cells. The physical interaction of NEU2 and GBA3 was confirmed by biochemical analyses and, consistent with this observation, the stabilization of NEU2 activity in the presence of GBA3 was evident both *in cellulo* and *in vitro*. Our results thus demonstrate an additional function of GBA3 in the catabolism of sialyl FNGs in the cytosol of mammalian cells.

## 2. Results

### 2.1. Involvement of GBA3 in the Catabolic Pathways for Sialyl-FNGs in the Cytosol of MKN45 Cells

**Figure 1:**
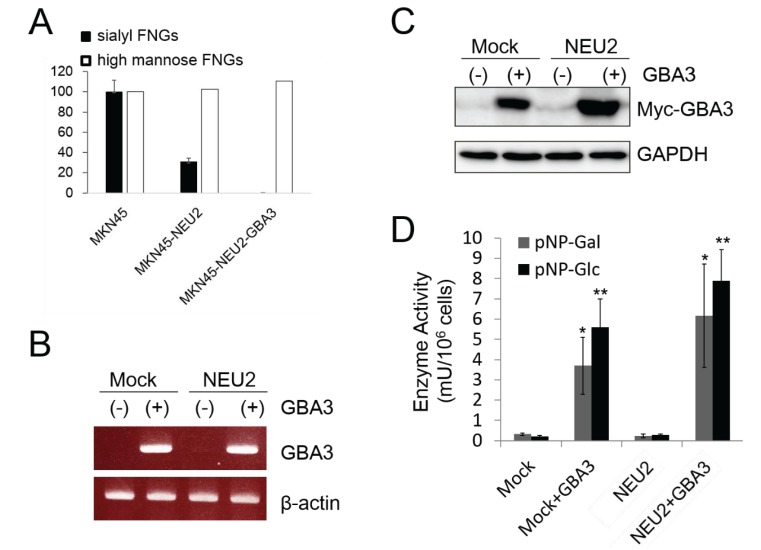
Expression of GBA3 in MKN45-Mock cells and MKN45-NEU2 cells. (**A**) Quantitation of sialyl FNG (Neu5Acα2-6Galβ1-4GlcNAcβ1-2Manα1-3Manβ1-4GlcNAc) (closed bar; *n* = 3) and high mannose-type FNG (Man_5-6_GlcNAc) (open bar; *n* = 1), in MKN45, MKN45-NEU2, and MKN45-NEU2/GBA3 cells. Amount of the sialylglycan or the high mannose-type glycans in MKN45 cells was set to 100. (**B**–**D**) 1 μg of pCMV-Myc-GBA3 as well as the mock vector (pCMV-Myc-N, Takara Bio Inc., Otsu, Japan) was transfected into 1 × 10^6^ MKN45Mock cells or MKN45-NEU2 cells. Cells were harvested 48 h after transfection for RT-PCR (**B**), western blotting (**C**), or GBA3 enzymatic activity assay (**D**). In **B**, β-actin was detected as the loading control; in **C**, GBA3 expression in cells was detected by an anti-Myc antibody, while GAPDH serves as the loading control. Representative data from three independent experiments are shown; error bars in **D** represent the ± SD. *: *p* < 0.05, **: *p* < 0.001 against control (Mock or NEU2).

We previously reported that high levels of complex-type sialyl free *N*-glycans (sialyl FNGs) accumulate in the cytosol of gastric adenocarcinoma cell lines MKN45 and MKN7 [10]. The objective of this study was to provide deeper insights into the mechanism of how those glycans are catabolized in MKN45 cells. To clarify the catabolic pathways for the sialyl FNGs found in the cytosol of MKN45 cells, we examined the effect of the overexpression of cytosolic glycosidases, namely NEU2 (a cytosolic sialidase) and GBA3 (β-galactosidase/β-glucosidase), in the amount of sialyl FNGs. Consistent with the previous observation, the levels of sialyl FNGs were significantly reduced in MKN45 cells that stably expressed NEU2 (MKN45-NEU2) [10] (Figure 1A). Next we investigated the effect of GBA3 expression. The original rationale behind this approach is that, if GBA3 were involved in the catabolism of desialylated, galactose-terminated FNGs after the NEU2 action of sialylated FNGs, the overexpression of GBA3 could further facilitate the catabolism of desialylated FNGs.

To validate this hypothesis, MKN45-Mock or MKN45-NEU2 cells were further transfected with Myc-tagged GBA3 or the vector control. As shown in Figure 1, we successfully obtained GBA3-expressing MKN45 cells in Mock (MKN45-GBA3) and NEU2-overexpressing cells (MKN45-NEU2/GBA3), as evidenced by RT-PCR analysis (Figure 1B) as well as by western blot analysis (Figure 1C). Furthermore, we detected GBA3 enzymatic activity in the cytosol of GBA3-expressing cells using pNP-β-Gal as well as pNP-β-Glc as substrates (Figure 1D). This broad substrate specificity of GBA3 is consistent with previously reported observations [16,19].

Having confirmed the enzymatic activity of expressed GBA3 proteins, we next examined the amount of sialyl FNGs in GBA3/NEU2-expressing cells. Unexpectedly, the co-expression of NEU2 and GBA3 resulted in a further decrease in sialyl FNG levels, to the point where they were barely detectable (Figure 1A for quantitation of the relative amount of Neu5Acα2-6Galβ1-4GlcNAcβ1-2Manα1-3Manβ1-4GlcNAc, the most abundant sialyl FNGs). In sharp contrast, no drastic change was observed in high mannose-type glycan levels (Figure 1A). These results clearly indicate that GBA3 is somehow involved in the efficient catabolism of sialyl FNGs in MKN45 cells.

**Figure 2:**
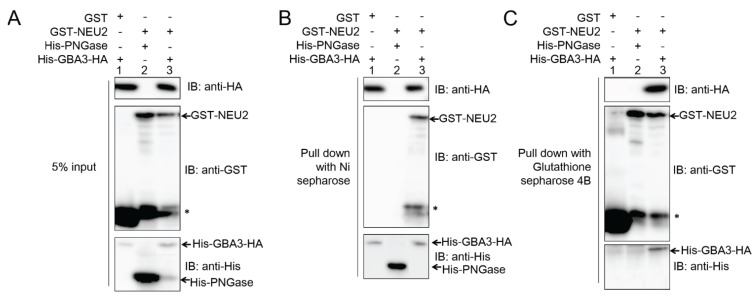
Co-precipitation assay of recombinant His-GBA3-HA and GST-NEU2. 500 μL of purified recombinant His-GBA3-HA (1.5 mg/mL) and recombinant GST-NEU2 (1.5 mg/mL) were incubated together overnight at 4 °C. As the negative control, His-GBA3-HA was mixed with GST alone and GST-NEU2 was mixed with His-PNGase, respectively. Five percent of input was shown by western blotting (**A**). The mixtures were then pulled down with 20 μL Ni-Sepharose™ (GE Healthcare Japan, Tokyo, Japan) 6 Fast Flow (**B**) or Glutathione-Sepharose™ (GE Healthcare Japan) 4B (**C**), and proteins that were bound to the resin were boiled in sample buffer, subjected to SDS-PAGE and western blotting. An anti-HA antibody was used to detect His-GBA3-HA; an anti-GST antibody was used to detect GST and GST-NEU2; an anti-His antibody was used to detect His-GBA3-HA and His-PNGase. Representative data from three independent experiments were shown. Asterisk (*) indicates the proteolytic fragments of GST-NEU2 which co-purify with the intact protein.

### 2.2. GBA3 Binds to NEU2 to Form a Complex

The fact that GBA3 somehow caused a drastic reduction of sialyl FNG levels led us to initially hypothesize that GBA3 in the cells may be involved as a cytosolic β-galactosidase in the catabolism of desialylated FNGs that are formed by the action of NEU2. However, despite repeated attempts, we were not able to detect any enzyme action of GBA3 towards FNGs or PA-labeled glycans of desialylated, complex-type FNGs (Li Wang and Tadashi Suzuki, RIKEN, Wako, Japan; Unpublished data, 2014). We therefore investigated whether the presence of GBA3 somehow affected the activity of NEU2, especially since it was previously reported that NEU2 undergoes lysosomal degradation through the autophagy process [20]. In an initial attempt, we examined whether the GBA3 physically interacts with NEU2. To this end, we expressed epitope-tagged GBA3 (His-GBA3-HA) and NEU2 (GST-NEU2) in *E. coli*, and the expressed proteins were purified using Ni-Sepharose and glutathione-Sepharose, respectively. The purified recombinant proteins were then incubated together overnight, and proteins were precipitated either with Ni-Sepharose or GST-sepharose, to determine if two proteins can be co-precipitated together. The protein input in the extract is shown in Figure 2A. As shown in Figure 2B, as the result of pull-down with Ni-Sepharose, GST-NEU2 was found to be co-precipitated (Figure 2B, middle panel; lane 3). In sharp contrast, GST (control) was not co-precipitated with His-GBA3-HA (Figure 2B, middle panel; lane 1) or His-PNGase (control) cannot be co-precipitated with GST-NEU2 (Figure 2B, middle panel; lane 2), further suggesting the specific binding between GBA3 and NEU2. Similar results are obtained by pull-down with glutathione-Sepharose (Figure 2C), and specific co-precipitation was observed between GST-NEU2 and His-GBA3-HA. The above collective results indicate the occurrence of a specific interaction between GBA3 and NEU2 proteins, as confirmed by a co-precipitation analysis.

**Figure 3:**
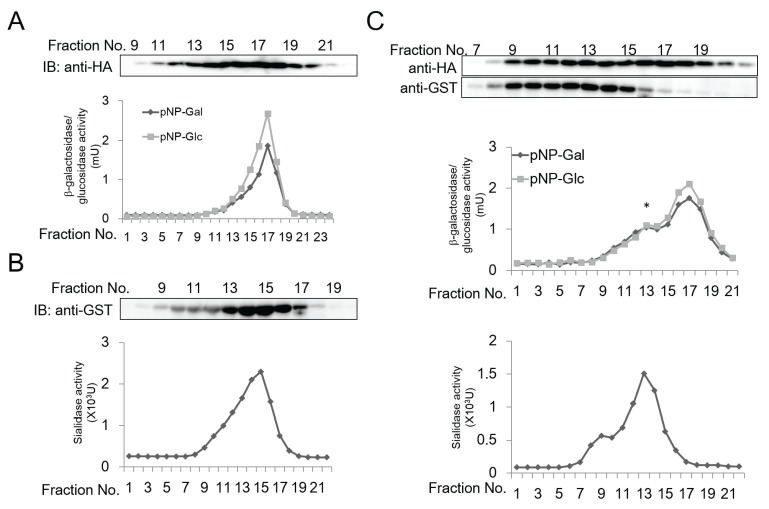
Gel filtration assay for recombinant His-GBA3-HA and GST-NEU2. (**A**) 500 μL of purified recombinant His-GBA3-HA (1.5 mg/mL) was subjected to a gel filtration assay and 0.9 mL fractions were collected. The components contained in an 0.8 mL aliquot of each fraction were precipitated with trichloroacetic acid (10%) and further subjected to western blotting for the detection of GBA3 by anti-HA antibody (top panel). 100 μL of fractions were assayed for enzymatic activity (bottom panel). (**B**) 500 μL of purified recombinant GST-NEU2 (1.5 mg/mL) was subjected to a gel filtration assay and 0.9 mL fractions were collected. The components contained in a 0.8 mL aliquot of each fraction were precipitated, and the resulting precipitates were subjected to western blotting for the detection of NEU2 by anti-GST antibody (top panel). 100 μL of fractions were assayed for sialidase activity (bottom panel). (**C**) 500 μL of purified recombinant His-GBA3-HA (1.5 mg/mL) and recombinant GST-NEU2 (1.5 mg/mL) were incubated together overnight at 4 °C, the mixture was then pulled down with Ni-Sepharose™ 6 Fast Flow. Proteins bound to the resin were analyzed by Sephacryl S-300 column. Fractions of 0.9 mL were collected. 0.7 mL of each fraction was precipitated subjected to western blotting for detection of GBA3 by anti-HA antibody and NEU2 by anti-GST antibody (top panel). Each 100 μL of fractions were assayed for GBA3 enzymatic assay (middle panel) and sialidase activity (bottom panel). Asterisk indicates the new GBA3 activity peak appeared upon incubation with NEU2. Representative data from three independent experiments were shown.

To further confirm the existence of a physical interaction between GBA3 and NEU2 to form a complex, a gel-filtration assay was carried out. When each protein was analyzed independently on a Sephacryl S-300 gel filtration column, GBA3 was eluted in fraction No. 17 as judged by a western blot analysis (Figure 3A, top) as well as an enzymatic activity assay (Figure 3A, bottom). On the other hand, NEU2 was eluted in fraction No. 15, as judged by western blot analysis (Figure 3B, top) and a sialidase assay (Figure 3B, bottom). Next, the two proteins were mixed together overnight, and GBA3 was pulled down by Ni-Sepharose and subjected to a gel-filtration assay monitored by an anti-HA western blot (Figure 3C, top panel) or an enzymatic activity assay (Figure 3C, middle panel). Upon co-incubation, a new peak for GBA3 at fraction No. 13 was observed. Since the pull-down was carried out using Ni-Sepharose, the protein appearing in fraction No. 17 should represent the free GBA3 protein, which could also be isolated by immunoprecipitation.

When the elution profile of NEU2 was examined using the same chromatography, the peaks for sialidase were shifted from the original elution profile (Figure 3B) and a new peak emerged at fraction No. 13 (Figure 3C, bottom panel), where an activity peak for GBA3 was also observed (Figure 3C, middle panel, peak indicated by asterisk). These results collectively suggest that the new peak at Fraction No. 13 represents a GBA3-NEU2 complex. While the new sialidase peaks at fraction No. 9 (the void fraction of this column) were also observed (Figure 3C, bottom panel), an additional peak corresponding to β-galactosidase/β-glucosidase activities was not observed in this fraction (Figure 3C, middle panel), despite the fact that both proteins were eluted in the same fraction (Figure 3C, top panel). These results suggest that the GBA3 in this fraction was enzymatically inactive, and the NEU2 observed in this fraction may correspond to aggregates.

**Figure 4:**
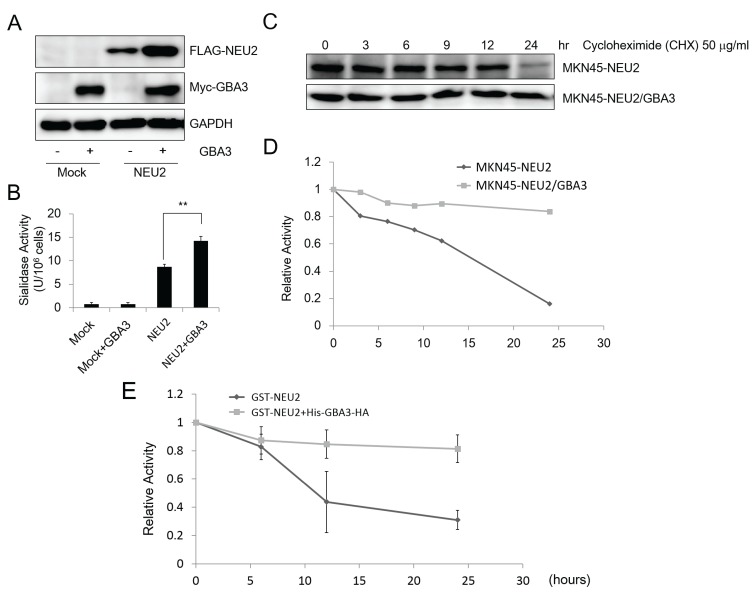
NEU2 was stabilized in the presence of GBA3. First, 1 μg of pCMV-Myc-GBA3 as well as the mock vector (pCMV-Myc-N, Clontech) was transfected into 1 × 10^6^ MKN45-Mock and MKN45-NEU2 cells. After a 48 h incubation, the cells were harvested, and were subjected to various analyses as follows: (**A**) western blotting for detection of NEU2 by anti-FLAG or GBA3 by anti-Myc antibody. GAPDH; as the loading control. (**B**) Sialidase (NEU2) activity assays of samples obtained in (**A)**. **: *p* < 0.001. (**C**) Cycloheximide decay assay. Cycloheximide was added to the 1 × 10^6^ of MKN45-NEU2 or MKN45-NEU2/GBA3 cells, and then cells were harvested at the indicated time points. Samples were analyzed by western blotting for analysis of NEU2 by anti-FLAG antibody. (**D**) Quantitation data for (**C**). The amount of protein at 0 time was set to 1. (**E**) Effect of GBA3 on the *in vitro* stability of NEU2. For this, 100 μL of purified GST-NEU2 (1.5 mg/mL) mixed with recombinant 100 μL of His-GBA3-HA (1.5 mg/mL) were incubated at 4 °C for the indicated times, and samples were subjected to a sialidase assay. For control incubation, 100 μL of buffer (25 mM MES buffer containing 100 mM NaCl (pH 6.0)) was added to the 100 μL of purified GST-NEU2. Activity of NEU2 at 0 time point was set to 1. Data shown represent data from three independent experiments. Error bars represent the ± SD.

### 2.3. GBA3 Co-Transfection Stabilized NEU2 Protein and Increased NEU2 Activity both *in Cellulo* and in Vitro Experiments

As shown in Figure 2 and Figure 3, our biochemical analyses using recombinant proteins strongly indicated the existence of a physical interaction between GBA3 and NEU2 *in vitro*. In addition to these analyses, we also noted that the amount of NEU2 protein was dramatically increased when co-expressed with GBA3 in MKN45 cells (Figure 4A), even when the same transfection conditions were applied. The increase in NEU2 expression was also confirmed by a sialidase assay (Figure 4B). Based on these results, we hypothesized that GBA3 stabilizes the NEU2 protein *in cellulo*. To validate this hypothesis, cycloheximide-decay experiments were carried out to test the stability of NEU2 in MKN45 cells with or without the co-expression of GBA3. As shown in Figure 4C,D, the NEU2 protein was time-dependently degraded in the absence of GBA3 while the stability of NEU2 was substantially enhanced in the presence of GBA3 protein. Sialidase (NEU2) activity was also found to be stabilized *in vitro* when co-incubated with His-GBA3-HA (Figure 4E). These results clearly suggest that the GBA3 protein stabilizes NEU2 proteins through physical interactions between the two molecules.

## 3. Discussion

In this study, we clearly demonstrate the existence of a physical interaction between GBA3 and NEU2 proteins by a series of *in cellulo* and *in vitro* analyses. It appears that GBA3 stabilizes the NEU2 proteins through binding to them, and thereby the catabolism of sialyl FNGs was greatly enhanced in MKN45-NEU2/GBA3 cells. While our initial expectation was that GBA3 may be involved in the degradation of desialylated FNGs in the cytosol after the action of NEU2, we failed to provide experimental evidence to show that GBA3 can directly act on desialylated FNGs *in vitro*, even in the presence of the NEU2 protein. This is puzzling, since GBA3 is known to show a broad substrate specificity towards various β-glycosides [14,16], and it may still be possible that additional factor(s) may be required for GBA3 to act on desialylated FNGs *in vivo*. Consequently, the detailed molecular mechanism responsible for the catabolism of desialylated complex-type glycans in the cytosol of mammalian cells still remains unclarified.

The physical interaction between NEU2 and GBA3 is reminiscent of the formation of a complex between lysosomal sialidase, NEU1, and β-galactosidase, together with other proteins such as the protective protein cathepsin A (PPCA) and GalNAc-6-sulfate sulfatase [21,22,23,24,25,26,27]. While a direct interaction between NEU2 and GBA3 was observed in this study, more proteins may be involved in the formation of a complex between NEU2 and GBA3 *in vivo*. Incidentally, cytosolic sialidase was reported to have a broad elution profile (estimated molecular weight of 60,000–200,000) when a crude extract fraction was examined [28]. This variety may be derived from the formation of complexes of NEU2 proteins *in vivo*.

How general is the NEU2-mediated degradation of sialyl FNGs that occurs in the cytosol of human cells? Since the expression of the *NEU2* gene was extremely low or undetectable in many human tissues/cells [29], the issue of whether or not it represents a major degradation pathway for sialyl FNGs remains to be clarified. It has been shown that sialyl FNGs in the cytosol can be catabolized by starvation-induced autophagy [11], possibly by delivering cytosolic FNGs to the lysosomes for degradation. It is therefore possible that the autophagy process may play a key role in the degradation of sialyl FNGs. It should also be noted that, while cytosolic β-glycosidase activity can be found in various mammals including the guinea pig [17,30], human [19,31], pig [32], or calf [33], such activity has not been reported in mice. Furthermore, while the *GBA3* gene was identified in humans [15,16] or the guinea pig [14], the obvious gene orthologue was not found in mice (*M. musculus*). Consequently, it may be possible that the conditions for the catabolism of cytosolic sialyl FNGs might be quite distinct between mammalian species. If so, further studies will be required to provide deeper insights into the details of the nonlysosomal catabolic pathway for sialyl FNGs in mammalian cells.

## 4. Material and Methods

### 4.1. Cell Culture

MKN45 cells were maintained in RPMI 1640 (Nacalai Tesque Co., Kyoto, Japan) with 10% fetal bovine serum and antibiotics (100 units/mL penicillin G; 100 ng/mL streptomycin, Nacalai Tesque Co.).

### 4.2. Western Blotting

Cells (1 × 10^6^) were washed twice with PBS (phosphate buffered saline), lysed in 500 μL of 2× SDS-PAGE sample buffer (8% SDS, 40% glycerol, 0.25 M Tris-HCl (pH 6.8), 2% BPB and 5% β-mercaptoethanol), and boiled for 10 min. Following SDS-PAGE, the gel was subjected to western blotting and visualized using a LAS3000 mini (Fujifilm Co., Tokyo, Japan) and Immobilon Western Reagents (Millipore). Antibodies used in the experiments were as follows: mouse monoclonal (M2) anti-FLAG antibody (dilution; 1:5000) was purchased from Sigma-Aldrich Co., LLC (St. Louis, MO, USA); the rabbit polyclonal anti-Myc antibody (1:5000), the mouse anti-GST antibody (1:5000), and the mouse anti-HA antibody (1:5000) were obtained from Santa Cruz biotechnology; the mouse anti-(His)_6_ antibody (1:500) was obtained from Roche; the mouse anti-GAPDH antibody (1:5000) was from Millipore. Secondary antibodies conjugated with horseradish peroxidase (anti-Rabbit IgG antibody (1:5000) and anti-Mouse IgG antibody (1:5000, except for the case with anti-GAPDH (1:10,000))) were purchased from Cell Signaling Technology, Inc. (Danvers, MA, USA).

### 4.3. RT-PCR

To examine *GBA3* mRNA expression via RT-PCR, cDNA was prepared from MKN45 cells as described previously [34]. The following two primers were used to assess *GBA3* expression: sense primer: 5'-ATGGCTTTCCCTGCAGGATT-3'; antisense primer: 5'-CTACAGATGTGCTTCAAGGC-3'. The primers for β-actin were: sense primer: 5'-AGAAAATCTGGCACCACACC-3'; antisense primer: 5'-CCATCTCTTGCTCGAAGTCC-3'. PCR products were resolved on a 2% agarose gel to detect the amplified DNA.

### 4.4. Extraction and PA-Labeling of Free Oligosaccharides Recovered from Cytosol

Individual aliquots of 1 × 10^8^ cells were washed twice with cold PBS, harvested, and lysed in 800 μL of lysis buffer (10 mM Hepes/NaOH buffer (pH 7.4), 5 mM DTT, 250 mM mannitol, 1 mM EDTA (pH 8.0), 1 × complete protease inhibitor cocktail (Roche Diagnostics K.K., Tokyo, Japan), and 1 mM Pefabloc (Roche Diagnostics K.K.)). The cell lysates were homogenized and centrifuged at 1000× *g* for 10 min at 4 °C to remove nuclei and unbroken cells. The supernatant was then centrifuged at 100,000× *g* for 1 h at 4 °C; the resulting supernatant was used as the cytosolic fraction. To precipitate proteins, 1.5 volumes of ethanol were added to the cytosolic fraction; each solution was mixed well and centrifuged at 17,000× *g* for 20 min at 4 °C. Each supernatant, which contained the free FNGs, was dried up to ~100 μL to remove the excess ethanol, and was desalted using a PD-10 column (GE Healthcare Japan) according to the manufacturer’s instructions. Detailed methods for PA-labeling as well as the removal of the excess reagent have been described previously [35,36], and the resulting sample was used for subsequent HPLC analysis.

### 4.5. HPLC Analysis

PA-labeled oligosaccharides were fractionated by various HPLC techniques. For anion exchange chromatography, a TSKgel DEAE-5PW column (7.5ϕ × 75 mm; Tosoh, Tokyo, Japan) was used, as described previously [11]. For analyzing sialyl FNGs, the following conditions were used: solvent A, 10% acetonitrile, 0.01% triethylamine; solvent B, 10% acetonitrile, 3% acetic acid, 7.4% triethylamine; and flow rate was 1.0 mL/min. Elution conditions were as follows: 0–10 min, 100% A; and 10–20 min, 15% solvent B. Peaks were monitored by fluorescence with λ_ex_ = 310 nm and λ_em_ = 380 nm. Sialyl FNGs that were sensitive to *A. ureafaciens* sialidase were collected and used for further analyses.

For analyzing desialylated PA-labeled glycans by size fractionation HPLC, a Shodex NH2P-50 4E column, in conjunction with a GL Sciences HPLC system (PU611 double pumps/CO630 column oven) and a fluorescence detector (LaChrom; Hitachi High-Technologies Co., Tokyo, Japan) were used. The elution was performed using two solvent gradients as follows: solvent A: 93% acetonitrile in 0.3% acetate (pH adjusted to 7.0 with ammonia); solvent B: 20% acetonitrile in 0.3% acetate (pH adjusted to 7.0 with ammonia). The gradient program (expressed as the percentage of solvent A): 0–5 min: isocratic 97%; 5–8 min: 97%–67%; 8–40 min: 67%–29%. Eluted compounds were detected by fluorescence with λ_ex_ = 310 nm and λ_em_ = 380 nm. Conditions for the separation of sialylated oligosaccharides on the amide-80 column were preformed, as reported previously [37]. Identification of PA-labeled glycans on ODS columns were performed as described previously [38].

### 4.6. Plasmid Transfection into Mammalian Cells

pCMV-Tag2-Flag-NEU2, which expresses N-terminal FLAG-tagged human NEU2 protein and its mock vector (pCMV-Tag2, Agilent Technologies, Santa Clara, CA, USA) [10] were transfected into 1 × 10^6^ MKN45 cells using FuGENE HD (Roche Diagnostics K.K.) or the Nucleofactor Transfection Kit (Amaxa; Lonza Japan, Tokyo, Japan) according to the manufacturer’s protocols. After 48 h, the medium was replaced with RPMI/10% FBS containing 200 μg/mL of G418 (Nacalai Tesque Co.) to select stable transfectants.

*GBA3* gene was amplified from cDNA prepared from human liver. Human liver mRNA was a kind gift from Drs. Kei-ichiro Inamori (Tohoku Pharm. Univ., Sendai, Japan) and Naoyuki Taniguchi (RIKEN, Wako, Japan), and cDNA was prepared using ReverTra Ace/Blend Taq (Toyobo Co. Ltd, Osaka, Japan) according to the vendor’s protocol. The primers used for the GBA3 amplification were as follows: forward primer: 5'-ACGCGTCGACCATGGCTTTCCCTGCAGGATT-3', reverse primer: 5'-ATAAGAATGCGGCCGCCTACAGATGTGCTTCAAGGC-3'. The fragment was cloned into TA cloning vector (Invitrogen, Life Technologies Japan, Tokyo, Japan). After the DNA sequence was confirmed, the SalI/NotI fragment was cloned into the equivalent site of pCMV-Myc-N vector (Takara Bio Inc.) to obtain pCMV-Myc-GBA3. pCMV-Myc-GBA3 or the mock vector (pCMV-Myc-N) was transfected into 1 × 10^6^ cells using the Nucleofactor Transfected Kit (Lonza Japan) according to the manufacturer’s protocols. Cells were harvested 48 h after transfection for further analyses.

### 4.7. E. coli Expression and Purification of Recombinant GBA3 and NEU2

For *E. coli* expression of recombinant GBA3, the coding sequence was amplified using pCMV-Myc-GBA3 as a template with the following primers: 5'-CACCATGGCTTTCCCTGCAGGATT-3' and 5'-CTACAGATGTGCTTCAAGGC-3', and cloned into Pdest™ 17 vector (Life Technologies Japan) for expression of N-terminal 6 × His tagged GBA3 (His-GBA3). The HA sequence was then inserted to the C-terminus of the sequence using the following primers: 5'-CGACGTCCCAGACTACGCTTAGTTGTACAAAGTGGTTGATTC-3' and 5'-CTGGGACGTCGTATGGGTACAGATGTGCTTCAAGGCCATTG-3', using the Quikchange site-directed mutagenesis (Agilent Technologies) according to the manufacturer’s protocol to construct pDEST17-His-GBA3-HA. The DNA sequence of the construct was confirmed by DNA sequencing analysis.

*E. coli* strain BL21(AI) cells were transformed with pDEST17-His-GBA3-HA and grown at 37 °C for 12 h with shaking in 500 mL of LB medium supplemented with 100 μg/mL of ampicillin. Then *L*-arabinose was added to a final concentration of 0.02% for 3 h with shaking at 25 °C to induce protein expression. Cells were harvested by centrifugation (5000× *g* for 10 min), washed twice with cold PBS, and suspended in 20 mL of extraction solution (1% Triton X-100 in PBS with 1× complete protease inhibitor cocktail (Roche Diagnostics K.K.) and 1 mM Pefabloc (Roche Diagnostics K.K.)). After sonication for 6 intervals (10 s each time), cell debris was removed by centrifugation (15,000× *g* for 30 min). A 4 mL aliquot of the obtained supernatant was applied to a 200 μL (bed volume) of Ni-Sepharose 6 Fast Flow (GE Healthcare Japan) column, and the column was washed with 10 bed volumes of 20 mM imidazole in 20 mM sodium phosphate/0.5 M NaCl buffer (pH 7.4). The proteins adsorbed to the resin were then eluted with 5 bed volumes of 500 mM imidazole in 20 mM sodium phosphate/0.5 M NaCl buffer (pH 7.4). The eluted fraction was dialyzed against 1 L of 25 mM MES buffer containing 100 mM NaCl (pH 6.0) and aliquots were stored at −80 °C for further analyses. For *E. coli* expression of recombinant NEU2, *E. coli* strain BL21(DE3) cells were transformed with pGEX4T-NEU2 [39] and grown at 37 °C for 12 h in 500 mL of LB medium supplemented with 100 μg/mL of Ampicillin with shaking. The pGEX4T-NEU2 was a generous gift from Dr. Soichi Wakatsuki (Stanford University, Stanford, CA, USA). IPTG was added to a final concentration of 0.05 mM for 12 h with shaking at 15 °C to induce protein expression. The procedure used to extract the protein was the same as above. For purification, 4 mL of supernatant obtained was applied to a 200 μL (bed volume) of Glutathione Sepharose™ 4B (GE Healthcare Japan) column. After washing the column with 10 bed volumes of PBS, the protein adsorbed to the column was eluted with 5 bed volumes of 10 mM glutathione in 50 mM Tris-HCl buffer (pH 8.0). Eluted fraction was dialyzed against 1 L of 50 mM MES buffer (pH 6.7) and aliquots were stored at −80 °C for further analyses. Before storage of purified GBA3 or NEU2, protein concentration was adjusted to 1.5 mg/mL.

### 4.8. Co-Precipitation Assay

To verify the interaction between NEU2 and GBA3 *in vitro*, a co-precipitation assay was carried out. In this assay, purified recombinants His-GBA3-HA and GST-NEU2 were mixed overnight at 4 °C. Aliquots of the mixture were then incubated with 20 μL of Ni Sepharose™ 6 Fast Flow (GE Healthcare Japan) or Glutathione Sepharose 4B (GE Healthcare Japan) for 1 h at 4 °C, and were spun down at 2300× *g* for 10 min. The resins containing protein complexes were washed three times with PBS and were suspended in sample buffer. As the negative control, His-GBA3-HA was mixed with GST alone and GST-NEU2 was mixed with (His)_6_-tagged yeast PNGase (His-PNGase; [40]), respectively. The mixtures were then pulled down with Ni-Sepharose™ 6 Fast Flow or Glutathione-Sepharose 4B. The resulting samples were subjected to SDS-PAGE and western blotting.

### 4.9. Gel Filtration Assay

Recombinant His-GBA3-HA and GST-NEU2 proteins were prepared in the presence of 1× complete protease inhibitor cocktail (Roche Diagnostics K.K.) and 1 mM Pefabloc (Roche Diagnostics K.K.). Aliquots of the two proteins were incubated together overnight at 4 °C. A 0.5 mL aliquot of each protein or mixture (1.5 mg/mL of each) was loaded on a Sephacryl S-300 column (GE Healthcare Japan; 1.5 × 50 cm) equilibrated with elution buffer (300 mM NaCl in 50 mM Tris-HCl, pH 7.5), respectively, and fractions of 0.9 mL were collected. Fractions were assayed for enzyme activity, and 0.8 mL of each fraction was precipitated with trichloroacetic acid (10%) and further subjected to SDS/PAGE and western blotting.

### 4.10. Cycloheximide-Decay Assay

For the cycloheximide-decay assay, MKN45-NEU2 cells or MKN45-NEU2/GBA3 cells were equally divided into 6 dishes (1 × 10^6^) and cycloheximide (Sigma) was added to each dish to a final concentration of 50 μg/mL before harvesting in 0.5 mL of SDS-PAGE sample buffer at the indicated time points. Samples were then analyzed by SDS/PAGE and western blotting.

### 4.11. Sialidase Assay

One hundred μL of cytosolic fractions of cells or 100 μL of diluted recombinant GST-NEU2 (1.5 μg/mL) were incubated with 100 μL of 0.1 mM 4MU-NeuAc (Sigma-Aldrich Co., LLC) in 0.2% Triton X-100/50 mM sodium citrate buffer (pH 6.0) at 37 °C for 4 h. After stopping the reaction by addition of 0.8 mL of 0.25 M glycine-NaOH buffer (pH 10.6), the fluorescence intensity was measured at 450 nm, with excitation at 365 nm by the Fluorescence spectrophotometer (F-4500, Hitachi High-Technologies Co.). One unit was defined as the amount required for to catalyze the release of 1 nmol of 4MU from 4MU-NeuAc in 1 h.

### 4.12. GBA3 Activity Assay

GBA3 enzymatic activity was measured on 5 mM *p*-nitro-phenyl-β-D-galactopyranoside (pNP-β-Gal; Sigma) and 5 mM *p*-nitro-phenyl-β-D-glucopyranoside (pNP-β-Glc; Sigma-Aldrich Co., LLC) in 20 mM sodium phosphate buffer (pH 6.5). Ten μL of cytosolic fractions of cells or 10 μL of diluted recombinant His-GBA3-HA (1.5 μg/mL) were incubated with 100 μL of pNP-β-Gal or pNP-β-Glc for 12 h at 37 °C. The reaction was stopped by adding 0.9 mL of a sodium borate solution (100 mM, pH 9.3). The absorbance was read at 450 nm by a microreader. One unit (U) of activity was defined as the amount of enzyme required to hydrolyze 1 μmol of substrate per hour.

## 5. Conclusions

We show that the amount of sialyl free *N*-glycans (FNGs) is dramatically reduced upon the co-expression of cytosolic sialidase NEU2 with cytosolic β-glycosidase GBA3 in human stomach cancer-derived MKN45 cells. The physical interaction between NEU2 and GBA3 was confirmed by co-precipitation analyses as well as gel filtration assays. The NEU2 protein was found to be stabilized in the presence of GBA3 both *in cellulo* and *in vitro*. Our results thus indicate that cytosolic GBA3 is likely involved in the catabolism of cytosolic sialyl free *N*-glycans, possibly by stabilizing the activity of the NEU2 protein.
